# Appraisal of Chitosan-Coated Lipid Nano-Combination with Miltefosine and Albendazole in the Treatment of Murine Trichinellosis: Experimental Study with Evaluation of Immunological and Immunohistochemical Parameters

**DOI:** 10.1007/s11686-024-00799-x

**Published:** 2024-03-15

**Authors:** Asmaa F. Ibrahim, Sahar M. Selim, Dalia A. Shafey, Dina M. Sweed, Shaimaa A. Farag, Marwa A. Gouda

**Affiliations:** 1 Clinical and Molecular Parasitology Department, National Liver Institute, Medical Campus, Menoufia University, Melig Road, Shebin El-Kom, 32511 Menoufia Governorate Egypt; 2 Pathology Department, National Liver Institute, Medical Campus, Menoufia University, Melig Road, Shebin El-Kom, Menoufia Governorate Egypt

**Keywords:** Trichinellosis, Chitosan-coated nanostructured lipid carriers, Miltefosine, GATA3, Glutathione peroxidase 1 (GPX1), Caspase-3

## Abstract

**Purpose:**

Resistance and adverse consequences of albendazole (ABZ) in treating trichinellosis urged demand for secure and effective new drugs. The current study aimed to assess the effect of chitosan-coated lipid nano-combination with albendazole and miltefosine (MFS) in treating experimental murine trichinellosis and evaluating pathological and immunological changes of trichinellosis.

**Materials and Methods:**

One hundred twenty Swiss albino mice were divided into six groups. Each group was subdivided into a and b subgroups based on the scarification time, which was 7- and 40-days post-infection (PI), respectively. The treatment efficacy was evaluated using parasitological, histopathological, serological (interleukin (IL)-12 and IL-4 serum levels), immunohistochemical (GATA3, glutathione peroxidase1 (GPX1) and caspase-3), and scanning electron microscopy (SEM) methods.

**Results:**

The most effective drug was nanostructured lipid carriers (NLCs) loaded with ABZ (G5), which showed the most significant reduction in adults and larval count (100% and 92.39%, respectively). The greatest amelioration in histopathological changes was reported in G4 treated with MFS. GATA3 and caspase-3 were significantly reduced in all treated groups. GPX1 was significantly increased in G6 treated with MFS + NLCs. The highest degenerative effects on adults and larvae by SEM were documented in G6.

**Conclusion:**

Loading ABZ or MFS on chitosan-coated NLCs enhanced their efficacy against trichinellosis. Although ABZ was better than MFS, their combination should be considered as MFS caused a significant reduction in the intensity of infection. Furthermore, MFS showed anti-inflammatory (↓GATA3) and antiapoptotic effects (↓caspase-3), especially in the muscular phase. Also, when loaded with NLCS, it showed an antioxidant effect (↑GPX1).

**Graphical abstract:**

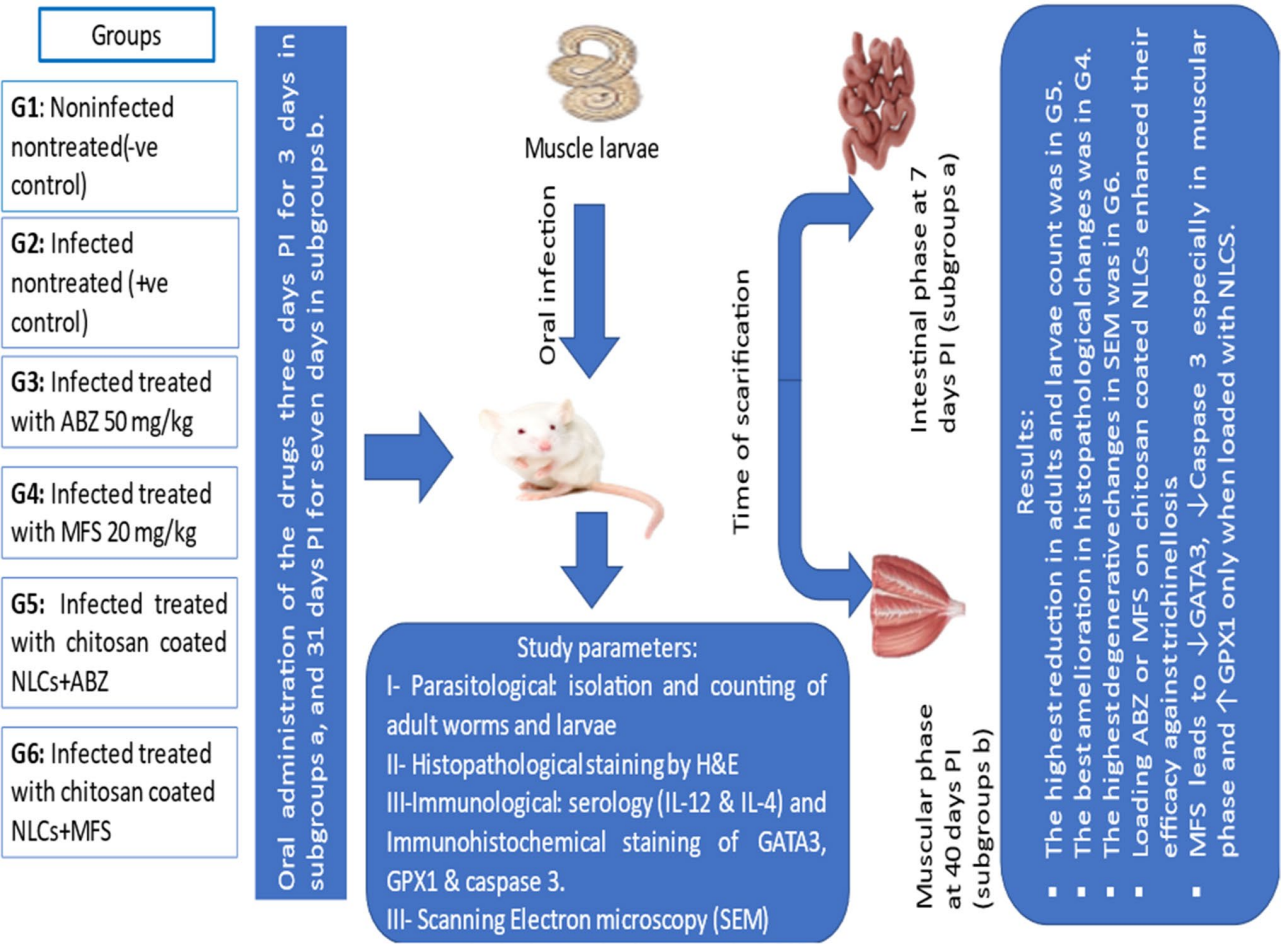

## Introduction

Trichinellosis is a public health hazard that affects not only human individuals but also an essential economic issue in animal and food safety [1]. Ingestion of infected animals’ raw or undercooked meat is the primary source of *Trichinella spiralis (T. spiralis)* transmission, which infects about 11 million people worldwide [2]. *T. spiralis* life cycle is distinguished by distinct enteral phase and parenteral phases, which can be categorized into migrating phase, wherein the larvae circulate within the bloodstream, and the muscular phase, characterized by the encapsulation of larvae in the skeletal muscles of the host [3, 4]. Most human infections are accidental, but severe complications such as myocarditis or encephalitis could happen, particularly in the elderly, and lead to death [5].

The immune response is initiated by stimulating T helper type 1 (Th1) cells during the intestinal stage. This is followed by a predominant T helper type 2 (Th2) response, characterized by the secretion of interleukins (IL) such as IL-4, IL-5, IL-9, IL-10, and IL-13. Additionally, this Th2 response leads to the production of immunoglobulin E (IgE) and the recruitment of eosinophils, basophils, and mast cells [6]. The muscular phase is characterized by T regulatory (Treg cells). The host’s immune response can be effectively modulated by the coordinated immune events mediated by Th2 and Treg cell populations [7]. GATA3 is a key factor in allergic responses and immune defense against worm infections [8]. It exhibits the ability to interact with the DNA sequence GATA, which is crucial for determining the phenotypic characteristics of Th0 cells, favoring their commitment to the Th2 lineage while inhibiting their differentiation into Th1 cells. Additionally, GATA3 promotes the production of IL-4, IL-5, and IL-13 from Th2 cells [9, 10].

The primary contributing factors to the pathology of *T. spiralis* are the mechanical injury induced by infection, inflammatory cells accumulation, and disruptions in the normal redox status [11]. The invasion of the host intestinal epithelium by infective larvae during the early stages leads to changes in the levels of pro-inflammatory cytokines and chemokines and alterations in the antioxidant capacity. These changes are implicated in the exacerbation of the underlying tissue damage [12]. The oxidant/antioxidant status is generally achieved through the equilibrium between the generation of reactive oxygen species (ROS) and reactive nitrogen species (RNS), as well as their elimination by antioxidants. Consequently, tissues frequently exhibit an augmented production of antioxidants in reaction to mild levels of oxidative stress [11].

Previous studies have demonstrated alterations in specific antioxidant enzymes levels, including superoxide dismutase (SOD), catalase (CAT), glutathione-S-transferase (GST), and glutathione peroxidase (GPX), within the muscle tissues of hosts during experimental trichinellosis [13, 14]. Furthermore, it was observed that there was an elevation in the occurrence of apoptotic events in the cells of the villus lamina propria during intestinal trichinellosis, as well as during the muscular phase when nurse cell formation takes place. The observed alterations are linked to an increase in the expression of apoptosis-related factors, including Bcl-2-associated protein X (BAX), Tumor necrosis factor-alpha (TNF-α), caspase-3, caspase-8, and caspase-9 [15, 16].

Albendazole (ABZ) is the drug of choice in treating trichinellosis [3, 17]. Despite its effectiveness, it is active against adult-stage and non-encysted muscle larvae only [11]. Moreover, its bioavailability is restricted because it is a poorly water-soluble and highly lipophilic drug with high reported resistance. Several methods were employed to improve the dissolution rate and, subsequently, oral absorption of ABZ [18–21].

Nanostructured lipid carriers (NLCs) were proposed as a potential solution to overcome the problem of poor drug entrapment, as suggested by Talegaonkar and Bhattacharyya [21]. The potential advantages of NLCs could be further enhanced through surface modification via chitosan coating, which may facilitate improved interaction with cellular membranes owing to the mucoadhesive characteristics of chitosan [3].

Miltefosine (MFS) is an alkyl phosphocholine initially developed as an anticancer agent. Verhaar et al. [22] say it has become the only effective oral therapy approved for treating human cutaneous and visceral leishmaniasis. It also exhibits anti-schistosomal properties and molluscicidal effects [23–25]. Moreover, it displays a diverse array of characteristics that are effective against metronidazole-resistant strains of *Trichomonas vaginalis* [26], *Giardia lamblia* (*G. lamblia*) [27], *Entamoeba histolytica* [28], various free-living amoebas [29], as well as *Toxoplasma gondii* (*T. gondii*) [30].

The present work aimed to assess the anti-*Trichinella,* and immunomodulatory effects of MFS and its nano-combination with chitosan-coated NLCs compared with ABZ against *T. spiralis* experimentally during both intestinal and muscular phases using parasitological, immunological, immunohistochemical, histopathological parameters, and scanning electron microscopy in evaluation.

## Materials and Methods

### Ethical Consideration

The Ethical Committee’s guidelines approved the experiments for handling and using laboratory animals. National Liver Institute No. 00342/2022.

### Experimental Design

Laboratory-bred Swiss albino female mice weighing about 20 g–25 g were housed in the Theodor Bilharz Research Institute (TBRI) biological unit, Giza, Egypt, at a temperature of 24 °C and fed a regular meal. Mice were classified into six groups (20 mice each), and each group (G) was further subdivided into two subgroups (subgroup a; for intestinal phase and subgroup b; for muscular phase), according to time of scarification [7 and 40 days post-infection (PI), respectively] [31]. G1; uninfected and untreated mice (negative control group), G2; infected untreated mice (positive control group), G3; infected and treated with ABZ, G4; infected and treated with MFS, G5; infected and treated with chitosan-coated NLCs loaded with ABZ, and G6; infected and treated with chitosan-coated NLCs loaded with MFS.

### Preparation of Chitosan-Coated Lipid Nano-Combination and Loading of the Drugs

Chitosan-coated NLCs were prepared at TBRI according to Eid et al. [3] as NLCs were created using micro-emulsification according to Joshi and Patravale [32] approach. All ingredients utilized to create the chitosan-coated NLCs were purchased from Sigma Aldrich, St. Louis, MO, USA, except for the glacial acetic acid, purchased from Pharaonic Ingredients Company, Cairo, Egypt. Following Das et al. [33], who employed the following equation, the drug loading was calculated as below:

$${\text{Drug~loading}}~\left( {{\text{mg/g}}} \right) = \frac{{{\text{Actual~drug~dosage}}}}{{{\text{Total~lipid~in~the~formulation}}}}.$$

The morphology and size measurements of nanoparticles and zeta potential were determined using transmission electron microscopy (TEM) (JEOL-JSM-1400 PLUS, Tokyo, Japan) and Zetasizer (Nano ZS, Malvern Instruments Ltd, Malvern, Worcestershire, United Kingdom) according to Eid et al. [3].

### Preparation of the Infective Inoculum

The *T. spiralis* isolates used in this work were kindly provided by the Medical Parasitology department, Tanta Faculty of Medicine, Tanta University, Egypt, and kept alive in TBRI’s laboratory by repeated passages in an animal model. The infective inoculum was prepared from laboratory-bred *T. spiralis*-infected mice, sacrificed five weeks PI. Their muscles were digested in 1% pepsin and 1% concentrated HCl in 200 ccs of distilled water. The mixture was then processed according to an earlier research protocol, and each mouse was orally infected with 200–300 T*. spiralis* larvae [20, 34].

### Drug Regimen and Administration

All drugs were administered orally as follows: in subgroups (a), drugs were administered three days PI for three consecutive days, while in subgroups (b), drugs were administered 31 days PI for seven consecutive days. Alzental tablets (200 mg/tablet) manufactured by Epico were used as a source of Albendazole. One tablet (200 mg) was dissolved in 50 ml of distilled water and given orally at 50 mg/kg/day [35] to G3. Miltefosine powder (1-hexadecyl phosphocholine, Chem-Impex International, New York, USA. Catalog No.# 21,603 was diluted in distilled water and given orally at a dose of 20 mg/kg/day [25] in G4. Chitosan-coated NLCs were prepared in a solution form in a dose of 250 μl/mouse, given orally, and loaded with either ABZ in G5 at a dose of 50 mg/kg or MFS in G6 at 20 mg/kg.

### Assessment of Infection and Drug Effects

For assessment of infection and drug effects, the studied subgroups (a and b) were subjected to the following:

#### Parasitological Study

1. Collection and counting of the adult worms of *T. spiralis* were done on the 7th day of PI in subgroups a [31].
2. Isolation and counting of muscular larvae were done on the 40th day PI in subgroups b. Larvae were isolated by artificial digestion of muscular tissues [4].

The percentage of reduction calculated according to Ashour et al. [36] as follows:

$$=100\times \frac{\left(\mathrm{mean\, No}.\mathrm{ recovered \,in \,controls }-\mathrm{ mean\, number\, recovered\, in\, treated \,mouse}\right)}{\mathrm{mean\, No}.\mathrm{ recovered\, in \,controls}}.$$

#### Histopathological Parameters

One centimeter was taken from the mid-intestinal region on the seventh day PI from all subgroups a. Tongue, diaphragm, and hind leg muscle samples from infected mice of subgroup b were collected, and all samples were processed histopathologically at the Pathology Department, National Liver Institute (NLI), Menoufia University. They were fixed in 10% neutral buffered formalin, dehydrated, immersed in xylene, and finally impregnated in paraffin [20]. Serial segments of 5 µm thickness stained with Hematoxylin and Eosin (H & E) were used to evaluate enteropathy and inflammatory response throughout the intestinal stage and the presence of larvae and degenerative inflammatory response during the muscular stage.

The number of larvae was scored histologically after examination of 10 low-power fields (LPF × 100) of each muscle segment: + 1 means =  < 5larvae, + 2 refers to between five and ten, and + 3 mean more than ten. To determine the severity of the inflammatory response, ten high-power fields (HPF × 400) in each intestinal section and ten low-power fields (LPF × 100) in each muscle tissue section were examined. The results were scored using a semi-quantitative system: 0 = Nil, + 1 = Few (up to 10 cells/HPF), + 2 = Moderate number (11–40 cells/HPF), and + 3 = Severe (more than 40 cells/HPF) [37, 38].

#### Immunological Parameters

##### Serology

Blood samples were collected at the 7th and 40th days PI; sera were separated and stored at−20°C until ELISA determined serum IL-12 and IL-4 levels. Mouse IL-12(p70) ELISA Kit PicoKine™ (Boster Biological Technology, Pleasanton CA, USA, Catalog #EK0422) and Mouse IL-4/Interleukin-4 ELISA Kit PicoKine^®^ (Boster Biological Technology, Pleasanton CA, USA, Catalog # EK0584). The instructions provided by the manufacturer conducted the ELISA techniques. The absorbance measurement for the serum samples was conducted within a 30-min timeframe, utilizing an ELISA microtiter plate readerat a specific wavelength of 450 nm.

##### Immunohistochemical Staining (IHC):

Paraffin sections from both intestinal and muscular phases underwent deparaffinization and rehydration. According to Mohammed et al. [39], the streptavidin–biotin-amplified system was used. The primary antibodies used were polyclonal glutathione peroxidase 1 (GPX1) diluted as 1:100 (Cat. #abx117034) (obtained from Abbexa, Milton, Cambridge, United Kingdom), monoclonal active caspase-3 diluted as 1:300 (Cat. #bsm33199M2) (obtained from Bioss Antibodies, Woburn, United States), and GATA Binding Protein 3 (GATA3) (Anti-GATA3 antibody [L50-823]) diluted as 1:2000 (Cat. #ab282110) obtained from Abcam, Cambridge, UK. 

Positive expression of GPX1 antibody showed cytoplasmic localization, caspase-3 antibody showed cytoplasmic and para nuclear localization, while GATA3 showed nuclear localization. The slides were assessed microscopically in HPF under (× 400) magnification by examining 20 HPF areas of intestinal and muscular sections of each examined animal and evaluated for the following parameters: Staining status: Positive cells for immunostain show brownish cytoplasmic and some nuclear staining with variable intensities graded as + 1 (mild), + 2 (moderate), and + 3 (strong). Histoscore (H-score) was applied to evaluate the studied section according to where both the intensity and percentage of positivity were considered using the following formula [40]: H-score =  + 1x% of mildly stained cells + 2x% moderately stained cells + 3x% of strongly stained cells. H-score ranged from 0 to 300.

#### Scanning Electron Microscopic Study (SEM) (Adult and Larvae)

Under a binocular microscope, adults of the intestinal phase and larvae of the muscular phase of the worms were extracted using a Pasteur pipette. They were immediately transferred into a fresh fixation solution of 2.5% glutaraldehyde (w/v) in 0.1 M sodium cacodylate at pH 7.2, and prepared for SEM study, which was carried out in an electron microscope unit, faculty of medicine, Tanta University, where they were observed by Jeol scanning electron microscope (Jeol Corp., Mitaka, Japan) [41].

### Statistical Analysis

The SPSS (Statistical Package for Social Science) program, version 20 Armonk, NY: IBM Corp., tabulated and analyzed the collected data. Mean, SD, and percentages served as the representation for descriptive statistics. The Chi-square test and the analysis of variance (ANOVA) were analytical statistics. The post hoc study evaluated the relationship between each pair of groups to determine any significant associations. If the P (probability) value was less than 0.05, it was deemed statistically significant.

## Results

### Mortality Incidence Among Studied Groups

There were two deaths in each 2b, 5b, and 6b subgroups but only one death in each 3b and 4b subgroups.

### Parasitological Results

Regarding the mean number of adult worms and encysted larvae, there was a statistically significant decrease in all treatment groups compared to the positive control group (*P* < 0.0001). The most significant reduction in the mean number of adult worms and encysted larvae was observed after treatment with chitosan-coated NLCs loaded with ABZ (G5), the reduction percentage was 100% and 92.39%, respectively. Followed by treatment with ABZ only (G3) as the percentage of reduction was 95.76% and 79.35%, respectively. Chitosan-coated NLCs were better than the ABZ suspension alone, either in intestinal or muscular phases. However, this enhancement caused by loading ABZ with NLCs only significantly reduces the larval count in the muscular phase (*P* < 0.05). Treatment with MFS either alone or loaded with chitosan-coated NLCs significantly reduced the count of larvae and adults in comparison to G2; the reduction was enhanced with loading with NLCs. Treatment with MFS alone caused a reduction in the adult and larval count at 33.17% and 57.76%, respectively, while loading MFS with chitosan-coated NLCs resulted in a reduction at 44.31% and 72.43%, respectively (Table 1).

**Table 1:** Comparison of the adult count and larval count between the controls and treated groups in the intestinal and muscular phases

	Adults count		Reduction%	ANOVA	*P* value Post hoc analysis
	Mean	SD			
Intestinal phase					
G1a; Negative control (*N* = 10)	0	0	––-	*P* = < 0.0001^**^	*P*1 = < 0.0001^**^ *P*2 = 0.878 *P*3 = < 0.0001^**^ *P*4 = 1 *P*5 = < 0.0001^**^ *P*6 = < 0.0001^**^ *P*7 = < 0.0001^**^ *P*8 = < 0.0001^**^ *P*9 = < 0.0001^**^ *P*10 = < 0.0001^**^ *P*11 = 0.878 *P*12 = < 0.0001^**^ *P*13 = < 0.0001^**^ *P*14 = 0.016^*^ *P*15 = < 0.0001^**^
G2a; Infected untreated (positive control) (*N* = 10)	82.60	9.640	0%		
G3a; Infected treated by ABZ (*N* = 10)	3.50	2.369	95.76%		
G4a; Infected treated by MFS (*N* = 10)	55.20	7.068	33.17%		
G5a; Infected treated by chitosan-coated NLCs + ABZ (*N* = 10)	0	0	100%		
G6a; Infected treated by chitosan-coated NLCs + MFS (*N* = 10)	46.00	9.262	44.31%		
Muscular phase					
G1b; Negative control (*N* = 10)	0	0	––-	*P* = < 0.0001^**^	*P*16 = < 0.0001^**^ *P*17 = < 0.0001^**^ *P*18 = < 0.0001^**^ *P*19 = 0.019^*^ *P*20 = < 0.0001^**^ *P*21 = < 0.0001^**^ *P*22 = < 0.0001^**^ *P*23 = < 0.0001^**^ *P*24 = < 0.0001^**^ *P*25 = < 0.0001^**^ *P*26 = 0.002^*^ *P*27 = 0.327 *P*28 = < 0.0001^**^ *P*29 = < 0.0001^**^ *P*30 = < 0.0001^**^
G2b; Infected untreated (positive control) (*N* = 8)	95218.75	10260.393	0%		
G3b; Infected treated by ABZ (*N* = 9)	19666.67	3535.534	79.35%		
G4b; Infected treated by MFS (*N* = 9)	40222.22	8273.116	57.76%		
G5b; Infected treated by chitosan-coated NLCs + ABZ (*N* = 8)	7250.00	2492.847	92.39%		
G6b; Infected treated by chitosan-coated NLCs + MFS (*N* = 8)	26250.00	6430.952	72.43%		

*P* values for subgroups a	*P* values for subgroups b
P1 relation between groups 1a & 2a	P16 Relation between groups 1b & 2b
P2 relation between groups 1a & 3a	P17 Relation between groups 1b & 3b
P3 relation between groups 1a & 4a	P18 Relation between groups 1b & 4b
P4 relation between groups 1a & 5a	P19 Relation between groups 1b & 5b
P5 relation between groups 1a & 6a	P20 Relation between groups 1b & 6b
P6 relation between groups 2a & 3a	P21 Relation between groups 2b & 3b
P7 relation between groups 2a & 4a	P22 Relation between groups 2b & 4b
P8 relation between groups 2a & 5a	P23 Relation between groups 2b & 5b
P9 relation between groups 2a & 6a	P24 Relation between groups 2b & 6b
P10 relation between groups 3a & 4a	P25 Relation between groups 3b & 4b
P11 relation between groups 3a & 5a	P26 Relation between groups 3b & 5b
P12 relation between groups 3a & 6a	P27 Relation between groups 3b & 6b
P13 relation between groups 4a & 5a	P28 Relation between groups 4b & 5b
P14 relation between groups 4a & 6a	P29 Relation between groups 4b & 6b
P15 relation between groups 5a & 6a	P30 Relation between groups 5b & 6b

Bold values are the significant *P* values to distinguish them from non significant *P* values

Significant *P* value is less than 0.05.^*^significant, ^**^highly significant

### Histopathological Results

**Fig. 1:**
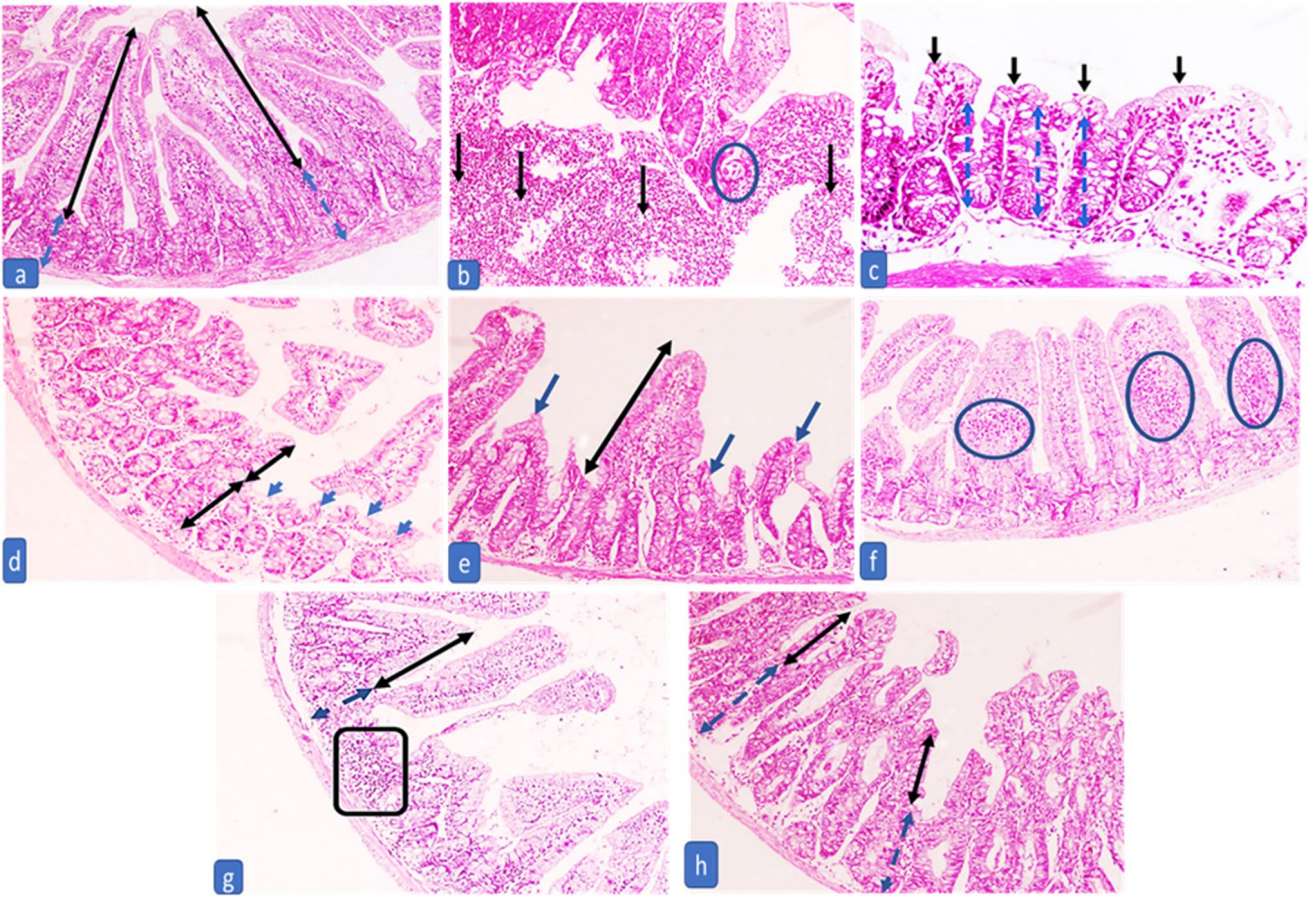
The intestinal phase of subgroup 1a shows normal villi and crypts in the mucosa **a**. Subgroup 2a shows encysted *Trichinella* larvae (circle) associated with a decrease of villous/crypt ratio and dense inflammation with lymphoid aggregates (arrows) **b** and marked blunting of villi (black arrows) and a decrease in villous crypt ratio (blue arrows) **c**. The treated subgroup with ABZ (3a) shows a moderate decrease in villous/crypt ratio (black arrow) with blunting of villi (blue arrows) and moderate edema **d**. The treated subgroup with MFS (4a) shows a normal villous/crypt ratio (black arrow) while a moderate decrease in other villi (blue arrows), associated with mild crypt hyperplasia and edema **e** plus mild inflammation rich in eosinophils with sheets formation (circles) **f**. Treated subgroup 5a (chitosan-coated NLCS loaded with ABZ) shows a mild decrease in villous/crypt ratio (black arrows), mild crypt hyperplasia (blue arrows), mild inflammation with eosinophils and lymphoid aggregate formation (box) plus mild edema **g**. Treated subgroup 6a (chitosan-coated NLCS loaded with MFS) shows a mild decrease in villous/crypt ratio (black arrows), mild crypt hyperplasia (blue arrows), mild inflammation, and mild edema **h** (H&E × 40)

**Fig. 2:**
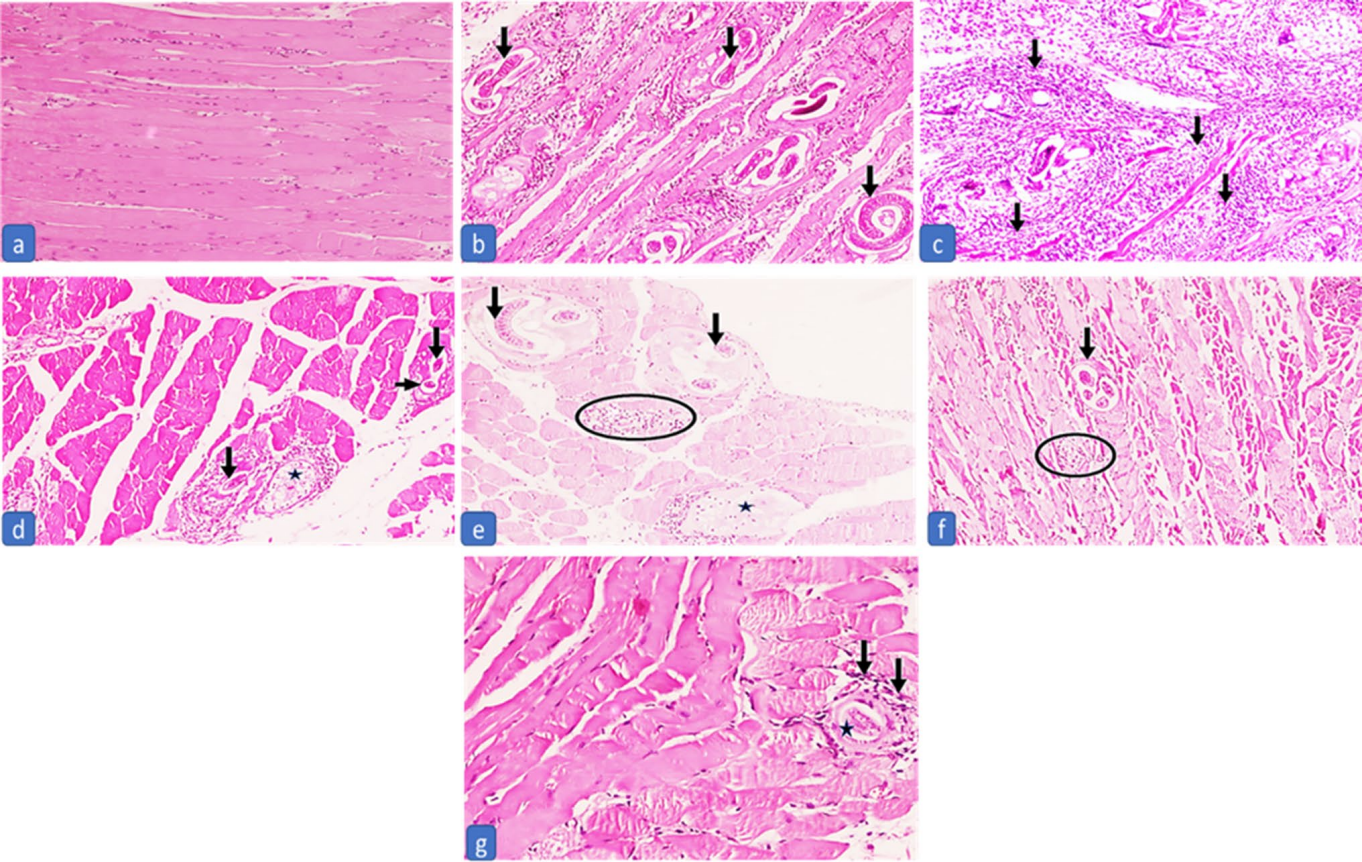
The muscular phase of the negative control group shows normal muscle without any inflammation or larvae **a**. Infected untreated subgroup 2b shows a large density of encysted *Trichinella* sp. larvae (+ 3) associated with moderate inflammation (+ 2), mild fragmentation of the internal structures (black arrows) **b**, and marked inflammation (+ 3) surrounding the larvae, dissecting and destructing the muscle (black arrows) **c**. Subgroup 3b shows a moderate density of encysted *Trichinella* sp. larvae (+ 2) associated with mild inflammation (+ 1) and severe fragmentation of the internal structures (black arrows) with occasional empty capsules (asterisk) **d**. Subgroup 4b shows a moderate density of encysted *Trichinella* sp. larvae (+ 2) associated with mild inflammation (+ 1) (circle) and average fragmentation of the internal structures (black arrows) and empty capsule (asterisk) **e**. Subgroup 5b shows a low density of encysted *Trichinella* sp. larvae (+ 1) with moderate fragmentation of the internal structures (black arrow) and little inflammation (+ 1) (circle) **f**. Subgroup 6b shows a low density of encysted *Trichinella* sp. larvae (+ 1) with severe fragmentation of the internal structures (asterisk) and mild inflammation (+ 1) (black arrows) **g** (H&E × 100)

In the intestinal phase, examination of the infected control (G2) revealed moderate to severe inflammation, edema, blunting of intestinal villi, and ulceration (Fig. 1b and c). There was a significant amelioration of histopathological changes and inflammation in all treated groups during both phases compared to the positive control group (G2) except in G3 (infected treated with ABZ) regarding the degree of inflammation in muscular phase as P21 > 0.05 (Tables 2 and 3 Figs. 1 and 2). In the intestinal phase, marked improvement of inflammation and amelioration in pathological changes was observed in the treated group with MFS (G4) followed by treated groups with NLCS (G5&6). Subgroup 3a treated with ABZ recorded the least improvement in pathological changes (Fig. 1). Muscles of the positive control group showed an increased intensity of encysted *T. spiralis* larvae, each surrounded by a thick intact capsule with a moderate to severe inflammatory infiltrate (Fig. 2b and c). The highest reduction in the number of encysted larvae with moderate to severe degeneration was observed in treated subgroup 5b with NLCs + ABZ, followed by treated subgroup 3b with ABZ **(**Table 3 Fig. 2**)**. However, the highest reduction in inflammatory changes was shown in treated subgroups with MFS as 4b and 6b (Table 2).G4, MFS treated, exhibited better histopathological outcomes compared to the remaining groups during both stages of infection, followed by chitosan-coated NLCS loading drugs either ABZ (G5) or MFS in (G6) (Tables 2 and 3).

**Table 2:** Comparison of the different degrees of inflammation between the controls and the treated groups in the intestinal and muscular phases

Intestinal phase		Degree of inflammation				Chi-square test	Multiple comparisons	*P*-value
		0	+1	+2	+3			
G1a; Negative control (*N* = 10)						*P* = < 0.0001^**^	X1 = 20 X2 = 20 X3 = 20 X4 = 20 X5 = 20 X6 = 8.33 X7 = 20 X8 = 13.7 X9 = 13.7 X10 = 6.66 X11 = 1.97 X12 = 1.97 X13 = 2.22 X14 = 2.22 X15 = 0	*P*1 = < 0.0001^**^ *P*2 = < 0.0001^**^ *P*3 = < 0.0001^**^ *P*4 = < 0.0001^**^ *P*5 = < 0.0001^**^ *P*6 = 0.016^*^ *P*7 = < 0.0001^**^ *P*8 = 0.001^*^ *P*9 = 0.001^*^ *P*10 = 0.010^*^ *P*11 = 0.16 *P*12 = 0.160 *P*13 = 0.136 *P*14 = 0.136 *P*15 = 1
No		10	0	0	0			
%		100%	0%	0%	0%			
G2a; Infected untreated (positive control) (*N* = 10)								
No		0	0	7	3			
%		0%	0%	70%	30%			
G3a; Infected treated by ABZ (*N* = 10)								
No		0	5	5	0			
%		0%	50%	50%	0%			
G4a; Infected treated by MFS (*N* = 10)								
No		0	10	0	0			
%		0%	100%	0%	0%			
G5a; Infected treated by chitosan-coated NLCs + ABZ (*N* = 10)								
No		0	8	2	0			
%		0%	80%	20%	0%			
G6a; Infected treated by chitosan-coated NLCs + MFS (*N* = 10)								
No		0	8	2	0			
%		0%	80%	20%	0%			
Muscular phase								
G1b; Negative control (*N* = 10)						*P* = < 0.0001^**^	X16 = 18 X17 = 19 X18 = 19 X19 = 18 X20 = 18 X21 = 3.95 X22 = 11.2 X23 = 7.11 X24 = 10.2 X25 = 3.88 X26 = 1.19 X27 = 4.48 X28 = 1.43 X29 = 0.018 X30 = 1.067	*P*16 = < 0.0001^**^ *P*17 = < 0.0001^**^ *P*18 = < 0.0001^**^ *P*19 = < 0.0001^**^ *P*20 = < 0.0001^**^ *P*21 = 0.138 *P*22 = 0.004^*^ *P*23 = 0.029^*^ *P*24 = 0.006^*^ *P*25 = 0.143 *P*26 = 0.549 *P*27 = 0.106 *P*28 = 0.232 *P*29 = 0.893 *P*30 = 0.302
No		10	0	0	0			
%		100%	0%	0%	0%			
G2b; infected-untreated (positive control) (N=8)								
No		0	0	5	3			
%		0%	0%	62.5%	37.5%			
G3b; Infected treated by ABZ (*N* = 9)								
No		0	3	5	1			
%		0%	33.3%	55.6%	11.1%			
G4b; Infected treated by MFS (*N* = 9)								
No		0	7	2	0			
%		0%	77.8%	22.2%	0%			
G5b; Infected treated by chitosan-coated NLCs + ABZ (*N* = 8)								
No		0	4	4	0			
%		0%	50%	50%	0%			
G6b; Infected treated by chitosan-coated NLCs + MFS (*N* = 8)								
No		0	6	2	0			
%		0%	75%	25%	0%			

*P* values for subgroups a	*P* values for subgroups b
X1, P1 relation between groups 1a & 2a	X16, P16 Relation between groups 1b & 2b
X2, P2 relation between groups 1a & 3a	X17, P17 Relation between groups 1b & 3b
X3, P3 relation between groups 1a & 4a	X18, P18 Relation between groups 1b & 4b
X4, P4 relation between groups 1a & 5a	X19, P19 Relation between groups 1b & 5b
X5, P5 relation between groups 1a & 6a	X20, P20 Relation between groups 1b & 6b
X6, P6 relation between groups 2a & 3a	X21, P21 Relation between groups 2b & 3b
X7, P7 relation between groups 2a & 4a	X22, P22 Relation between groups 2b & 4b
X8, P8 relation between groups 2a & 5a	X23, P23 Relation between groups 2b & 5b
X9, P9 relation between groups 2a & 6a	X24, P24 Relation between groups 2b & 6b
X10, P10 relation between groups 3a & 4a	X25, P25 Relation between groups 3 b & 4b
X11, P11 relation between groups 3a & 5a	X26, P26 Relation between groups 3 b & 5b
X12, P12 relation between groups 3a & 6a	X27, P27 Relation between groups 3 b & 6b
X13, P13 relation between groups 4a & 5a	X28, P28 Relation between groups 4 b & 5b
X14, P14 relation between groups 4a & 6a	X29, P29 Relation between groups 4 b & 6b
X15, P15 relation between groups 5a & 6a	X30, P30 Relation between groups 5 b & 6b

Bold values are the significant *P* values to distinguish them from non significant *P* values

**Degree of inflammation:** 0 (no), + 1 (mild), + 2 (moderate), and + 3 (intense) inflammatory reaction, Significant *P* value is less than 0.05.^*^significant, ^**^highly significant

**Table 3:** Comparison of larval deposition intensity score, different degrees of degradation of the internal structure of the larva, and muscle state among the controls and treated groups in the muscle phase

Muscular phase	Larval deposition intensity score				Chi-square test	*P* value
	0	+ 1	+ 2	+ 3		
G1b; Negative control (*N* = 10)					X1 = 18 X2 = 19 X3 = 19 X4 = 18 X5 = 18 X6 = 8.57 X7 = 3.74 X8 = 11.2 X9 = 9.14 X10 = 2.33 X11 = 4.73 X12 = 2.04 X13 = 5.24 X14 = 2.72 X15 = 1.06	*P*1 = < 0.0001^**^ *P*2 = < 0.0001^**^ *P*3 = < 0.0001^**^ *P*4 = < 0.0001^**^ *P*5 = < 0.0001^**^ *P*6 = 0.014^*^ *P*7 = 0.154 *P*8 = 0.004^*^ *P*9 = 0.01^*^ *P*10 = 0.311 *P*11 = 0.030^*^ *P*12 = 0.152 *P*113 = 0.73 *P*14 = 0.256 *P*15 = 0.302
No	10	0	0	0		
%	100%	0%	0%	0%		
G2b; infected untreated (positive control) (*N* = 8)						
No	0	0	3	5		
%	0%	0%	37.5%	62.5%		
G3b; Infected treated by ABZ (*N* = 9)						
No	0	2	7	0		
%	0%	22.2%	77.8%	0%		
G4b; Infected treated by MFS (*N* = 9)						
No	0	2	5	2		
%	0%	22.2%	55.6%	22.2%		
G5b; Infected treated by chitosan-coated NLCs + ABZ (*N* = 8)						
No	0	6	2	0		
%	0%	75%	25%	0%		
G6b; Infected treated by chitosan-coated NLCs + MFS (*N* = 8)						
No	0	4	4	0		
%	0%	50%	50%	0%		

	Degradation of the internal structure of the larva				Chi-square test	*P* value
	Absent	Mild	Moderate	Severe		
Muscular phase						
G1b; Negative control (*N* = 10)					X1 = 18 X2 = 19 X3 = 19 X4 = 18 X5 = 18 X6 = 0 X7 = 1.4 X8 = 4.8 X9 = 4.7 X10 = 1.43 X11 = 4.76 X12 = 4.8 X13 = 5.6 X14 = 4.107 X15 = 2.06	*P*1 = < 0.0001^**^ *P*2 = < 0.0001^**^ *P*3 = < 0.0001^**^ *P*4 = < 0.0001^**^ *P*5 = < 0.0001^**^ *P*6 = 1 *P*7 = 0.232 *P*8 = 0.091 *P*9 = 0.092 *P*10 = 0.232 *P*11 = 0.092 *P*12 = 0.091 *P*13 = 0.61 *P*14 = 0.128 *P*15 = 0.356
No	10	0	0	0		
%	100%	0%	0%	0%		
G2b; Infected untreated (positive control) (*N* = 8)						
No	0	4	4	0		
%	0%	50%	50%	0%		
G3b; Infected treated by ABZ (*N* = 9)						
No	0	4	5	0%		
%	0%	44.4%	55.5%	0%		
G4b; Infected treated by MFS (*N* = 9)						
No	0	2	7	0		
%	0%	22.2%	77.8%	0%		
G5b; Infected treated by chitosan-coated NLCs + ABZ (*N* = 8)						
No	0	1	3	4		
%	0%	12.5%	37.5%	50%		
G6b; Infected treated by chitosan-coated NLCs + MFS (*N* = 8)						
No	0	1	4	3		
%	0%	12.5%	50%	37.5%		

X1, P1 relation between groups 1b & 2b	X9, P9 Relation between groups 2b & 6b
X2, P2 relation between groups 1b & 3b	X10, P10 Relation between groups 3 b & 4b
X3, P3 relation between groups 1b & 4b	X11, P11 Relation between groups 3 b & 5b
X4, P4 relation between groups 1b & 5b	X12, P12 Relation between groups 3 b & 6b
X5, P5 relation between groups 1b & 6b	X13, P13 Relation between groups 4 b & 5b
X6, P6 relation between groups 2b & 3b	X14, P14 Relation between groups 4 b & 6b
X7, P7 relation between groups 2b & 4b	X15, P15 Relation between groups 5 b & 6b
X8, P8 relation between groups 2b & 5b	

Larval deposition intensity score: 0 (no), + 1 (mild), + 2 (moderate), and + 3 (intense), Significant *P* value is less than 0.05.^*^significant, ^**^highly significant

### Immunological Results

#### Serum Levels of IL-12 and IL-4

In the intestinal phase, a significant elevation of serum IL-12 level in the positive control group was observed compared to the negative control group, followed by a reduction of its level in the muscular phase. All treated groups showed a significant reduction in the level of IL-12 compared to the positive control group either in intestinal or muscular phases. Chitosan-coated NLCs loaded with MFS (subgroup 6a) showed the highest reduction in the intestinal phase. Also, it was noted that the reduction was more in groups treated with NLCs than in corresponding groups treated with crude drugs. While in the muscular phase, the same occurred except that the highest reduction occurred in subgroup 5b treated with NLCs with ABZ **(**Table 4**)**.

**Table 4:** Comparison of the IL-12 and IL-4 levels between the controls and treated groups in the intestinal and muscular phases

	IL-12		ANOVA	Post hoc analysis	IL-4		ANOVA	Post hoc analysis
	Mean	SD			Mean	SD		
Intestinal phase								
G1a; Negative control (*N* = 10)	170.50	8.683	*P* = < 0.0001^**^	P1 = < 0.0001^**^ P2 = < 0.0001^**^ P3 = < 0.0001^**^ P4 = < 0.0001^**^ P5 = < 0.0001^**^ P6 = < 0.0001^**^ P7 = < 0.0001^**^ P8 = < 0.0001^**^ P9 = < 0.0001^**^ P10 = < 0.0001^**^ P11 = < 0.0001^**^ P12 = < 0.0001^**^ P13 = < 0.0001^**^ P14 = < 0.0001^**^ P15 = 0.006^*^	29.90	2.767	*P* = < 0.0001^**^	P1 = 0.512 P2 = 0.774 P3 = 0.270 P4 = < 0.0001^**^ P5 = < 0.0001^**^ P6 = 0.016^*^ P7 = 1.000 P8 = < 0.0001^**^ P9 = < 0.0001^**^ P10 = 0.005^*^ P11 = < 0.0001^**^ P12 = < 0.0001^**^ P13 = < 0.0001^**^ P14 = < 0.0001^**^ P15 = < 0.0001^**^
G2a; Infected untreated (positive control) (*N* = 10)	500.70	14.461			27.50	2.563		
G3a; Infected treated by albendazole (*N* = 10)	427.60	16.015			31.80	1.900		
G4a; Infected treated by miltefosine (*N* = 10)	464.80	6.143			27.00	3.245		
G5a; Infected treated by chitosan-coated NLCs + albendazole (*N* = 10)	315.60	2.633			45.70	2.669		
G6a; Infected treated by chitosan-coated NLCs + miltefosine (*N* = 10)	298.90	10.816			39.40	2.875		
Muscle phase								
G1b; Negative control (*N* = 10)	170.50	8.683	*P* = < 0.0001^**^	P16 = < 0.0001^**^ P17 = < 0.0001^**^ P18 = < 0.0001^**^ P19 = < 0.0001^**^ P20 = < 0.0001^**^ P21 = < 0.0001^**^ P22 = < 0.0001^**^ P23 = < 0.0001^**^ P24 = < 0.0001^**^ P25 = < 0.0001^**^ P26 = < 0.0001^**^ P27 = < 0.0001^**^ P28 = < 0.0001^**^ P29 = 0.913 P30 = 0.395	29.90	2.767	*P* = < 0.0001^**^	P16 = 0.202 P17 = < 0.0001^**^ P18 = < 0.0001^**^ P19 = 0.190 P20 = < 0.0001^**^ P21 = 0.224 P22 = < 0.0001^**^ P23 = < 0.0001^**^ P24 = < 0.0001^**^ P25 = < 0.0001^**^ P26 = 0.002^*^ P27 = 0.327 P28 = < 0.0001^**^ P29 = < 0.0001^**^ P30 = 0.152
G2b; Infected untreated (positive control) (*N* = 8)	433.75	14.646			33.50	2.563		
G3b; Infected treated by albendazole (*N* = 9)	393.00	6.614			37.11	1.900		
G4b; Infected treated by miltefosine (*N* = 9)	337.11	4.167			44.56	3.245		
G5b; Infected treated by chitosan-coated NLCs + albendazole (*N* = 8)	213.00	4.811			55.63	2.669		
G6b; Infected treated by chitosan-coated NLCs + miltefosine (*N* = 8)	221.12	4.016			51.63	2.875		

*P* values for subgroups a	*P* values for subgroups b
X1, P1 relation between groups 1a & 2a	X16, P16 Relation between groups 1b & 2b
X2, P2 relation between groups 1a & 3a	X17, P17 Relation between groups 1b & 3b
X3, P3 relation between groups 1a & 4a	X18, P18 Relation between groups 1b & 4b
X4, P4 relation between groups 1a & 5a	X19, P19 Relation between groups 1b & 5b
X5, P5 relation between groups 1a & 6a	X20, P20 Relation between groups 1b & 6b
X6, P6 relation between groups 2a & 3a	X21, P21 Relation between groups 2b & 3b
X7, P7 relation between groups 2a & 4a	X22, P22 Relation between groups 2b & 4b
X8, P8 relation between groups 2a & 5a	X23, P23 Relation between groups 2b & 5b
X9, P9 relation between groups 2a & 6a	X24, P24 Relation between groups 2b & 6b
X10, P10 relation between groups 3a & 4a	X25, P25 Relation between groups 3 b & 4b
X11, P11 relation between groups 3a & 5a	X26, P26 Relation between groups 3 b & 5b
X12, P12 relation between groups 3a & 6a	X27, P27 Relation between groups 3 b & 6b
X13, P13 relation between groups 4a & 5a	X28, P28 Relation between groups 4 b & 5b
X14, P14 relation between groups 4a & 6a	X29, P29 Relation between groups 4 b & 6b
X15, P15 relation between groups 5a & 6a	X30, P30 Relation between groups 5 b & 6b

Bold values are the significant *P* values to distinguish them from non significant *P* values

Significant *P* value is less than 0.05.*Significant, ^**^highly significant

The serum level of IL-4 in the positive control group was not significantly different from the normal level when compared to the negative control group either in intestinal or muscular phases (*P* > 0.05), but when comparing the corresponding subgroups of positive control group (subgroups 2a and 2b, respectively), the level of IL-4 was increased significantly in muscular phase when compared to intestinal phase (*P* < 0.0001). In all treated groups there was a significant elevation in IL-4 level compared to the positive control group either in the intestinal phase (except subgroup 4a treated with MFS) or in the muscular phase (except subgroup 3b treated with ABZ), and the level of IL-4 was significantly increased in muscular phase than intestinal phase when comparing corresponding subgroups. G5 treated with NLCs with ABZ showed the highest enhancement of IL-4 either in intestinal or muscular phases **(**Table 4**)**.

#### Immunohistochemical Results

Concerning immunohistochemical results, the positive control group showed significantly increased expression of GATA3, GPX1, and caspase-3 than the negative control group either in intestinal or muscular phases P1 < 0.000. All treated groups showed a significant reduction of both GATA3 and caspase-3 (except in group 3 treated with ABZ as P3 > 0.05) than the positive control group either in intestinal or muscular phases. Also, groups treated with chitosan-coated NLCs (G5 and G6) showed enhanced reduction than corresponding groups treated by crude drugs only (G3 and G4, respectively). The lowest expression of GATA3 was noticed in groups treated with MFs. The lowest expression of caspase-3 in intestinal or muscular phases occurred in group 6 treated with chitosan-coated NLCs loaded with MFS **(**Tables 5 and 6**)**.

**Table 5:** Comparison of the GATA3 and GPX1 H-score between the controls and treated groups in the intestinal and muscular phases

	GATA3		ANOVA	Post hoc analysis	GPX1		Post hoc analysis
	Mean	SD			Mean	SD	
Intestinal phase							
G1a; Negative control (*N* = 10)	4.00	3.944	*P* = < 0.0001^**^	P1 = < 0.0001^**^ P2 = < 0.0001^**^ P3 = < 0.0001^**^ P4 = < 0.0001^**^ P5 = < 0.0001^**^ P6 = < 0.0001^**^ P7 = < 0.0001^**^ P8 = < 0.0001^**^ P9 = < 0.0001^**^ P10 = 0.002^*^ P11 = < 0.0001^**^ P12 = < 0.0001^**^ P13 = 0.998 P14 = 0.913 P15 = 0.998	20.50	14.615	P1 = 0.000^**^ P2 = 0.002^**^ P3 = 0.014^*^ P4 = < 0.0001^**^ P5 = < 0.0001^**^ P6 = 0.998 P7 = 0.892 P8 = 0.985 P9 = 0.001 P10 = 0.998 P11 = 0.774 P12 = < 0.0001^**^ P13 = 0.357 P14 = < 0.0001^**^ P15 = 0.014^*^
G2a; Infected untreated (positive control) (*N* = 10)	171.50	11.797			140.00	52.068	
G3a; Infected treated by ABZ (*N* = 10)	81.00	11.738			124.00	44.020	
G4a; Infected treated by MFS (*N* = 10)	54.00	26.331			108.00	60.516	
G5a; Infected treated by chitosan-coated NLCs + ABZ (*N* = 10)	50.00	9.428			162.00	34.897	
G6a; Infected treated by chitosan-coated NLCs + MFS (*N* = 10)	46.00	13.499			249.00	66.908	
Muscular phase							
G1b; Negative control (*N* = 10)	5.00	5.270	*P* = < 0.0001^**^	P16 = < 0.0001^**^ P17 = < 0.0001^**^ P18 = 0.105 P19 = 0.042^*^ P20 = 0.953 P21 = 0.002^*^ P22 = < 0.0001^**^ P23 = < 0.0001^**^ P24 = < 0.0001^**^ P25 = 0.278 P26 = 0.484 P27 = 0.002^*^ P28 = 1 P29 = 0.674 P30 = 0.442	6.00	6.992	P16 = 0.080 P17 = 0.261 P18 = < 0.0001^**^ P19 = < 0.0001^**^ P20 = < 0.0001^**^ P21 = 0.999 P22 = 0.131 P23 = < 0.0001^**^ P24 = < 0.0001^**^ P25 = 0.021^*^ P26 = < 0.0001^**^ P27 = < 0.0001^**^ P28 = < 0.0001^**^ P29 = 0.015^*^ P30 = 0.008^*^
G2b; Infected untreated (positive control) (*N* = 8)	105.00	22.039			45.63	14.745	
G3b; Infected treated by ABZ (*N* = 9)	59.44	28.553			37.22	13.017	
G4b; Infected treated by MFS (*N* = 9)	34.37	14.530			83.33	38.079	
G5b; Infected treated by chitosan-coated NLCs + ABZ (*N* = 8)	38.13	36.541			186.25	50.692	
G6b; Infected treated by chitosan-coated NLCs + MFS (*N* = 8)	16.11	17.204			132.50	19.821	

P values for subgroups a	P values for subgroups b
X1, P1 relation between groups 1a & 2a	X16, P16 Relation between groups 1b & 2b
X2, P2 relation between groups 1a & 3a	X17, P17 Relation between groups 1b & 3b
X3, P3 relation between groups 1a & 4a	X18, P18 Relation between groups 1b & 4b
X4, P4 relation between groups 1a & 5a	X19, P19 Relation between groups 1b & 5b
X5, P5 relation between groups 1a & 6a	X20, P20 Relation between groups 1b & 6b
X6, P6 relation between groups 2a & 3a	X21, P21 Relation between groups 2b & 3b
X7, P7 relation between groups 2a & 4a	X22, P22 Relation between groups 2b & 4b
X8, P8 relation between groups 2a & 5a	X23, P23 Relation between groups 2b & 5b
X9, P9 relation between groups 2a & 6a	X24, P24 Relation between groups 2b & 6b
X10, P10 relation between groups 3a & 4a	X25, P25 Relation between groups 3 b & 4b
X11, P11 relation between groups 3a & 5a	X26, P26 Relation between groups 3 b & 5b
X12, P12 relation between groups 3a & 6a	X27, P27 Relation between groups 3 b & 6b
X13, P13 relation between groups 4a & 5a	X28, P28 Relation between groups 4 b & 5b
X14, P14 relation between groups 4a & 6a	X29, P29 Relation between groups 4 b & 6b
X15, P15 relation between groups 5a & 6a	X30, P30 Relation between groups 5 b & 6b

Bold values are the significant *P* values to distinguish them from non significant *P* values

Significant *P* value is less than 0.05.^*^significant, ^**^highly significant

**Table 6:** Comparison of caspase-3 expression between the controls and the treated groups in the intestinal and muscular phases

	Caspase-3 expression				Chi-square test	Multiple comparisons	*P* value
	0	+1	+2	+3			
Intestinal phase							
G1a; Negative control (*N* = 10)					*P* = < 0.0001^**^	X1 = 20 X2 = 20 X3 = 16 X4 = 15.2 X5 = 14.6 X6 = 1.97 X7 = 7.6 X8 = 13 X9 = 14 X10 = 3.37 X11 = 8.33 X12 = 9.09 X13 = 2.27 X14 = 2.66 X15 = 0.22	*P*1 = < 0.0001^**^ *P*2 = < 0.0001^**^ *P*3 = 0.001^*^ *P*4 = 0.001^*^ *P*5 = 0.001^*^ *P*6 = 0.160 *P*7 = 0.022^*^ *P*8 = 0.001^*^ *P*9 = 0.001^*^ *P*10 = 0.185 *P*11 = 0.016^*^ *P*12 = 0.011^*^ *P*13 = 0.320 *P*14 = 0.264 P15 = 0.639
No	8	2	0	0			
%	80%	20%	0%	0%			
G2a; Infected untreated (positive control) (*N* = 10)							
No	0	0	2	8			
%	0%	0%	20%	80%			
G3a; Infected treated by ABZ (*N* = 10)							
No	0	0	5	5			
%	0%	0%	50%	50%			
G4a; Infected treated by MFS (*N* = 10)							
No	0	2	6	2			
%	0%	20%	60%	20%			
G5a; Infected treated by chitosan-coated NLCs + ABZ (*N* = 10)							
No	0	3	7	0			
%	0%	30%	70%	0%			
G6a; Infected treated by chitosan-coated NLCs + MFS (*N* = 10)							
No	0	4	6	0			
%	0%	40%	60%	0%			
Muscular phase							
G1b; Negative control (*N* = 10)					*P* = < 0.0001^**^	X16 = 18 X17 = 19 X18 = 19 X19 = 18 X20 = 18 X21 = 3.62 X22 = 12.9 X23 = 11.2 X24 = 16 X25 = 6.44 X26 = 4.38 X27 = 9.67 X28 = 0.476 X29 = 2.01 X30 = 3.69	P16 = < 0.0001^**^ *P*17 = < 0.0001^**^ *P*18 = < 0.0001^**^ *P*19 = < 0.0001^**^ P20 = < 0.0001^**^ P21 = 0.164 P22 = 0.002 *P*23 = 0.004^*^ *P*24 = < 0.0001^**^ *P*25 = 0.040^*^ *P*26 = 0.112 *P*27 = 0.008^*^ *P*28 = 0.490 *P*29 = 0.156 *P*30 = 0.055
No	10	0	0	0			
%	100%	0%	0%	0%			
G2b; Infected untreated (positive control) (*N* = 8)							
No	0	0	2	6			
%	0.0%	0.0%	25.0%	75.0%			
G3b; Infected treated by ABZ (*N* = 9)							
No	0	2	4	3			
%	0.0%	22.2%	44.4%	33.3%			
G4b; Infected treated by MFS (*N* = 9)							
No	0	7	2	0			
%	0.0%	77.8%	22.2%	0%			
G5b; Infected treated by chitosan-coated NLCs + ABZ (*N* = 8)							
No	0	5	3	0			
%	0.0%	62.5%	37.5%	0%			
G6b; Infected treated by chitosan-coated NLCs + MFS (*N* = 8)							
No	0	8	0	0			
%	0.0%	100%	0%	0%			

*P* values for subgroups a	*P* values for subgroups b
X1, P1 relation between groups 1a & 2a	X16, P16 Relation between groups 1b & 2b
X2, P2 relation between groups 1a & 3a	X17, P17 Relation between groups 1b & 3b
X3, P3 relation between groups 1a & 4a	X18, P18 Relation between groups 1b & 4b
X4, P4 relation between groups 1a & 5a	X19, P19 Relation between groups 1b & 5b
X5, P5 relation between groups 1a & 6a	X20, P20 Relation between groups 1b & 6b
X6, P6 relation between groups 2a & 3a	X21, P21 Relation between groups 2b & 3b
X7, P7 relation between groups 2a & 4a	X22, P22 Relation between groups 2b & 4b
X8, P8 relation between groups 2a & 5a	X23, P23 Relation between groups 2b & 5b
X9, P9 relation between groups 2a & 6a	X24, P24 Relation between groups 2b & 6b
X10, P10 relation between groups 3a & 4a	X25, P25 Relation between groups 3 b & 4b
X11, P11 relation between groups 3a & 5a	X26, P26 Relation between groups 3 b & 5b
X12, P12 relation between groups 3a & 6a	X27, P27 Relation between groups 3 b & 6b
X13, P13 relation between groups 4a & 5a	X28, P28 Relation between groups 4 b & 5b
X14, P14 relation between groups 4a & 6a	X29, P29 Relation between groups 4 b & 6b
X15, P15 relation between groups 5a & 6a	X30, P30 Relation between groups 5 b & 6b

Bold values are the significant *P* values to distinguish them from non significant *P* values

Degree of caspase-3 expression: 0 (no), + 1 (mild), + 2 (moderate), and + 3 (intense), Significant *P* value is less than 0.05.^*^significant, ^**^highly significant

Regarding GPX1 in the intestinal phase, the highest GPX1 H-score in treated groups occurred in subgroup 6a treated with chitosan-coated NLCs loaded with MFS. In the muscular phase, the highest score was in subgroup 5b treated with chitosan-coated NLCs loaded with ABZ, followed by subgroup 6b. Chitosan-coated NLCs either with ABZ or MFS increased the H-score than groups treated with crude drugs alone either in intestinal or muscular phases **(**Table 5**)**.

### Scanning Electron Microscopy

**Fig. 3:**
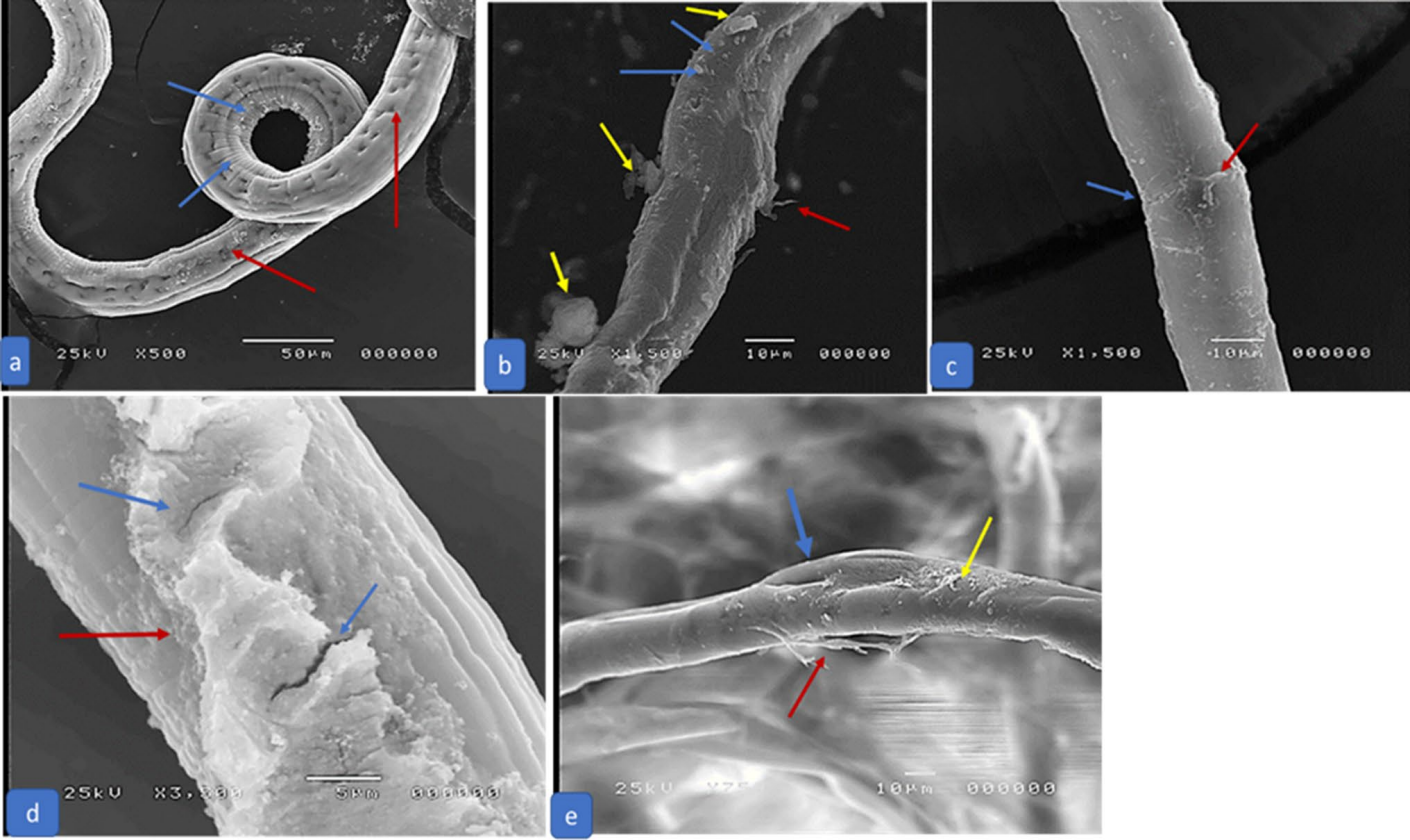
SEM of *T. spiralis* adult of an infected control mouse (G2) showing primary folds with large spacing. Transverse creases (blue arrows) and hypodermal glands opening (red arrows) **a**, infected treated with ABZ (G3) showing flattening of the worm, loss of normal annulations of the cuticle, large blebs (blue arrows), sloughing of parts of the cuticle (red arrows) and cauliflower masses (yellow arrows) **b**, infected treated with MFS (G4) showing loss of normal annulations of the cuticle, fissures (blue arrows) and sloughing of parts of the cuticle (red arrows) **c**, infected treated with chitosan-coated NLCs loaded with MFS (G6) showing sloughing of parts of the cuticle (red arrow) and fissuring of the cuticle (blue arrows) **d**, with loss of normal annulations of the cuticle, flattening of the worm, widening of the glandular opening (blue arrow), blebs on the cuticle (yellow arrow), and sloughing of parts of the cuticle (red arrow) **e **(colour figure online)

**Fig. 4:**
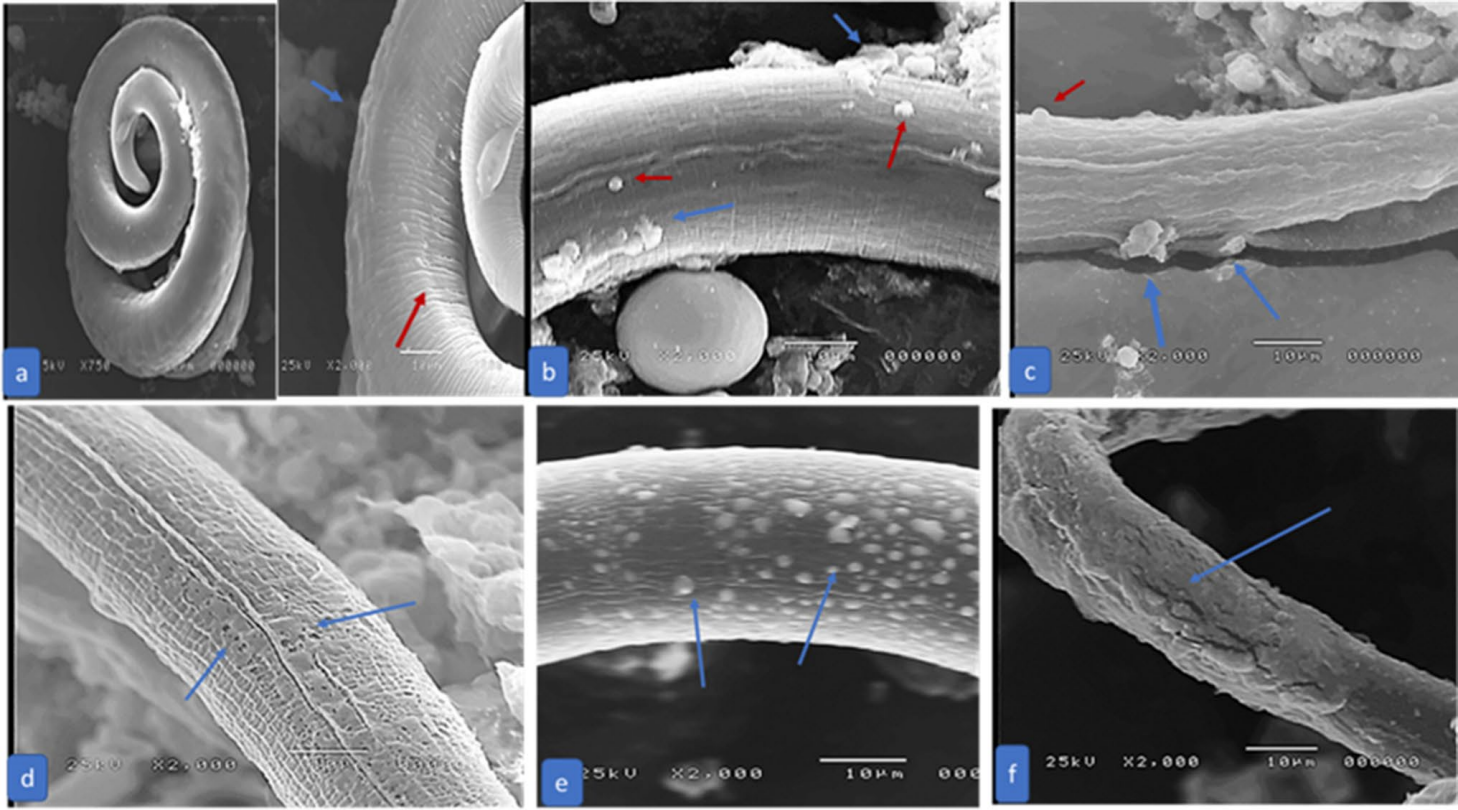
SEM of *T. spiralis* larva of an infected control mouse (G2) showed a typical coiled appearance with normal cuticle with fine longitudinal ridges (blue arrow) and transverse creases (red arrow) **a**, infected treated with ABZ (G3) showed blebs (red arrows) and cauliflower masses raised from the cuticle (blue arrows) **b**, infected treated with MFS (G4) showed blebs (red arrow), cauliflower masses (blue arrows) **c**, infected treated with NLCS and ABZ (G5) showed sloughing of the cuticle with erosions (blue arrows) **d**, infected treated with NLCs and MFS showed multiple large vesicles on the cuticle (blue arrows) **e**, with loss of normal transverse creases of the cuticle and several fissures in the cuticle (blue arrow) **f ** (colour figure online)

Remarkable degenerative changes in *T. spiralis* adult and larval structures were revealed in all treated groups except in treated subgroup 3b (ABZ). Due to a complete cure, no SEM was done in treated subgroup 5a (chitosan-coated NLCs loaded with ABZ). In subgroups a and b, the most remarkable changes were in the treated group with chitosan-coated NLCS loaded with MFS (G6). Also, NLCS enhanced the degenerative effect of loaded drugs, either ABZ or MFS **(**Figs. 3 and 4**)**.

## Discussion

Trichinellosis is a parasitic disease that ranks seventh among the top ten food-borne parasitic diseases. It holds substantial global medical importance as it influences muscle tissues and organs, consequently giving rise to profound health complications urging the need to develop and implement novel pharmaceutical interventions [42].

Loading ABZ with chitosan-coated NLCs in the present study enhanced its effect by significantly reducing worm counts in both phases. Our results agreed with Eid et al. [3], who found that loading the ABZ with chitosan-coated NLCs increased the reduction to 80.2% in the muscular phase. Also, a high reduction percentage was documented when ABZ was loaded with bovine serum albumin nanoparticles [20]. The mild documented effect of ABZ in an earlier study was referred to encystation of larvae in muscle tissues [43]. However, in the present study, we refer to the documented effect of a higher administered dose of ABZ and variation in scarification time.

To the best of our knowledge, this is the first study that used MFS in the treatment of experimental trichinellosis. MFS was used earlier in the treatment of *Schistosoma mansoni-*induced infection (*S. mansoni*) [23], and the efficacy was significantly improved with the use of lipid nano-capsules (LNCs) [44], which agreed with current study results.

The use of MFS showed the best result in the amelioration of histopathological changes, especially the reduction in the degree of inflammation; these results agreed with Eissa et al. [23], where the use of MFS either alone or loaded was accompanied by a statistically significant small granuloma and marked improvement in hepatic pathology [23, 44]. Similar results were also obtained when MFS was used in the treatment of *G. lamblia* and chronic toxoplasmosis infection experimentally [27, 30].

Histopathological changes resulted from the treatment with ABZ either alone or loaded with nanoparticles were similarly obtained by Eid et al. [3], as the administration of ABZ in the muscular phase of experimental trichinellosis resulted in intact larvae capsules inside muscles, accompanied by significant inflammatory infiltrations. However, the delivery of the drug in the coated NLCs form led to the degeneration of larvae, with less intense inflammatory changes observed.

During the early stage of intestinal infection by *T. spiralis*, there was a significant increase of Th1 cytokines such as IL-12, interferon-gamma (INF $\gamma )$, IL-1 $\beta$, and TNF-$\alpha$ associated with increased eosinophils and the development of intestinal pathology. IL-12 and INF-γ are vital as they participate in the polarization of the Th1-type immune response [42, 45]. All treated groups in the present study showed a significant reduction in the level of IL-12 in comparison to the positive control group either in intestinal or muscular phases, which coincides with Abd El-Hamed et al. [46], and Salama et al. [47] results, where a significant reduction in serum TNF-$\alpha$ was observed in all treated groups either with ABZ or herbal extracts at different PI periods. However, the significant reduction that occurred with ABZ either alone or loaded with NLCS did not concede with Wu et al. [48], where ABZ caused a non-significant reduction of the plasma level of IL-12 (*p* > 0.05) in *Echinococcus multilocularis*-infected mice. Also, the significant reduction that occurred with MFS either alone or combined with NLCs did not concede with Palić et al. [49, 50], who stated that MFS in the treatment of *Leishmania* targeted the Th-1 signaling, increasing the concentrations of pro-inflammatory Th-1 cytokines, such as IFN-γ and IL-12 and induced a shift of the macrophage phenotype, which is essential for the clearance of intracellular pathogens which we refer to the use of different infection models.

The increased level of IL-4 in the treated groups in this study was in harmony with Wang et al. [51]**,** who revealed that in rodent models of trichinellosis, a stable Th2 immune response is maintained during infection with *T. spiralis* after a short Th1 immune response. The role of Th2 cytokines such as IL-4, IL-5, and IL-13 in the intestinal phase is to stimulate IgE synthesis, inducing mast cell and eosinophil hyperplasia, triggering immediate hypersensitivity reactions, and promoting the *T. spiralis* expulsion from the intestine [52]. So, this explained that the increase in the level of IL-4 in treated groups was to promote the expulsion of the worms.

Regarding treatment with MFS, in the intestinal phase, there was no significant difference in IL-4 level in comparison to the positive control group, while in the muscular phase, there was a significant elevation in IL-4 level; these results are in contrast with Gupta et al. [53] as by 45 days post-treatment by MFS in experimental *Leishmania donovani, *there were significant decreases in IL-4, IL-10, and TGF-*β* in cured hamsters. This difference may be due to different drug regimens and different infections.

Expression of GATA3 in the positive control group was significantly enhanced either in intestinal or muscular phases than in the negative control group; these results agreed with Long et al*.* [54], as *T*. *spiralis* induced Th2 response in the mouse lung tissue, increased lung CD4 + T cells, GATA3, IL-4, IL-5, and IL-13 expression. Although different drugs used in this study caused a significant elevation of IL-4 in the positive control group, all drugs caused a significant reduction in GATA3 expression, and these results were in harmony with Hafez et al. [10], where a significant increase of GATA3 expression was detected in the infected control group. Vaccinated-challenged and *Punica-*treated groups showed a significant decrease. This was explained due to the anti-inflammatory effect of *Punica* that controls molecular mediators of inflammation such as nuclear factor kappa-B (NF-kB), MAPK (mitogen–activated protein kinase), and GATA3 by the bioactive components [55]. MFS shows anti-inflammatory effects in endothelial cells, suppressing vascular inflammation [56, 57]. So, the same mechanism might explain the results in this study. 

Treatment with ABZ caused a significant reduction in GATA3 and a non-significant reduction in GPX1 expression, which agreed to some extent with results documented by Wu et al. [48] and Kim et al. [58], where ABZ showed a non-significant reduction of messenger ribonucleic acid (mRNA) expressions of GATA3 transcription factor and the mRNA expression of the antioxidant enzymes catalase, GPX1, and GPX3 used in the treatment of experimental *Echinococcus multilocularis* and prostate cancer, respectively. This difference might be due to different drug regimens and infections.

Treatment with MFS alone in G4 caused a non-significant reduction of GPX1 compared to positive control G2 in the intestinal phase, while in the muscular phase, it caused non-significant enhancement. Loading the drug with chitosan-coated NLCs (G6) resulted in a significant enhancement either in the intestinal or muscular phases. This may be due to the direct effect of chitosan-coated NLCs on GPX1. These results were not agreed with Das et al. [59], where increased GPX and SOD activity levels in untreated visceral leishmaniasis patients were reversed after miltefosine chemotherapy.

Regarding the increase in caspase-3 expression either in intestinal or muscular phases in the positive control group, these results were consistent with Piekraska et al. [15]**;** Bruschi et al. [16] as an increase of apoptotic events described in cells of the villus lamina propria during intestinal trichinellosis, and upregulation of the expression of some apoptosis factors such as BAX, TNF-α, caspase-3, caspase-8, and caspase-9 associated with nurse cell formation during the muscular phase. 

Treatment with ABZ alone (G3) caused a non-significant difference in caspase-3 expression compared to the positive control group in either intestinal or muscular phases. However, loading ABZ with NLCs caused a significant reduction in caspase-3 expression. This may be due to the effect of NLCS itself on caspase-3. These results are in accordance with Petersen and Baird [60] as mebendazole and albendazole-induced classical apoptosis are characterized by caspase-3 activation, phosphatidylserine exposure, DNA fragmentation, mitochondrial membrane permeability, and ROS production. Similar results were obtained by Zhang et al. [61]. Treatment with MFS caused a significant reduction of caspase-3 expression in the positive control group in both intestinal and muscular phases, and this reduction was enhanced by loading MFS with NLCs. These results did not concede with Khademvatan et al. [62], as miltefosine induced dose-dependent death with features of apoptosis, including cell shrinkage, DNA laddering, and externalization of phosphatidylserine in both strains of *Leishmania major* and *Leishmania tropica*. Miltefosine has demonstrated activity against *Leishmania* parasites and neoplastic cells primarily due to its effects on apoptosis and disturbance of lipid-dependent cell signaling pathways [50]. This difference might be due to different infections and different drug regimens, as it was said that MFS induced a dose-dependent death. The maintenance of the external surfaces of helminths is crucial for their various physiological functions, including nutrient absorption, osmoregulation, immune protection, and structural support [63], so an SEM analysis study was conducted to evaluate the effect of different drugs on the cuticle of *T. spiralis.* Scanning electron microscopy showed that MFS-treated groups, either alone or loaded with NLCs, caused more significant destructive changes than treated groups with ABZ, especially in the muscular phase; the lipid structure of the epicuticular attached layer of *T. spiralis* infective larvae may explain this phenomenon [64] and the mechanism of action of MFS involves interaction with lipids including membrane lipids leading to the inhibition of lipid-dependent cell signaling pathways [22, 50]. So, it was clear that miltefosine attacks mainly the cell membrane. Similar results were obtained in *S. mansoni* and *G. lamblia* as MFS induced apparent and extensive damage to the tegumental surface of *S. mansoni* and severe morphological alterations to *G. lamblia* trophozoites, and it was explained that miltefosine could interact directly with the lipid bilayer and interfere with its biochemistry [23, 27]. Treatment with ABZ (G3) also showed severe destructive changes in the cuticle of *T. spiralis* adult but fewer effects on the muscle larvae. These results agreed with Abou Hussien et al. [63], where the flattening of adult worms, disappearance of typical transverse creases, and axial ridges were reported after in vitro incubation of adult and larval phases with ABZ. In the present study, loading drugs, either ABZ or MFS, with chitosan-coated NLCs caused more enhancement reduction of *T. spiralis* worms counts than corresponding groups treated by crude drugs either in intestinal or muscular phases. Eid and his colleagues [3] stated that NLCs exhibit greater efficacy than drug suspensions due to their propensity to adhere to the intestinal wall, potentially allowing for entry into the intervillous spaces. Additionally, the bio-adhesive properties of chitosan-coated NLCs may result in a higher local concentration of the drug on the membrane surface. Our hypothesis was to demonstrate the possible pathogenic role of miltefosine in the treatment of trichinellosis through three different pathways (inflammation (GATA 3), apoptotic (caspase-3), and oxidant/antioxidant pathways (GPX1)). In our study, MFS induced a significant effect on the elimination of inflammation and a significant antiapoptotic effect without any significant impact on the oxidant/antioxidant pathway (except when loaded on NLCs it caused a significant antioxidant effect). However, it caused significant oxidation when it was used in the treatment of leishmaniasis by Das et al. [3]

## Conclusion

In conclusion, the highest reduction in *T. spiralis* infection either in the intestinal or muscular phases was in the treated group with ABZ loaded on NLCs (G5), followed by crude ABZ (G3), while the best amelioration in histopathological changes and degree of inflammation was in the treated group with crude MFS (G4) followed by treated groups with NLCs either G5 or G6. This amelioration that occurred with MFS might be explained by its effect on IL-12, as it caused a significant reduction of its level and significant IL-4 elevation levels. Furthermore, MFS in this model caused a significant reduction of GATA3 (anti-inflammatory effect) and caspase-3 (antiapoptotic effect). It caused significant enhancement of GPX1 (antioxidant effect) only when loaded with NLCs. The most significant destructive changes, either in intestinal or muscular phases, occurred in G6 (treated with MFS + NLCS). Loading ABZ or MFS on chitosan-coated NLCs resulted in enhancing their efficacy. Although MFS results were not better than ABZ, MFS, either alone or loaded with chitosan-coated NLCs, could be considered a promising drug for the treatment of trichinellosis, especially in the muscular phase, or it could be used in combination with ABZ, to improve the effect of ABZ especially regarding the pathological changes. Nevertheless, additional research is required to determine the appropriate dosage and duration of therapy to attain greater outcomes. Also, further studies are required to study the effect of MFS and its combination with NLCs on the oxidant/antioxidant pathway and how to augment the antioxidant effect of MFS in the treatment of trichinellosis.

## Data Availability

The authors declare that all the information needed to substantiate the conclusions will be provided upon request.

## Abbreviations

ABZ: Albendazole
GPX1: Glutathione peroxidase 1
IHC: Immunohistochemical staining
IL-12: Interleukin 12
IL-4: Interleukin 4
MFS: Miltefosine
NLCs: Nanostructured lipid carriers
PI: Post-infection
SEM: Scanning electron microscopy
